# Surgical treatment of special type of adjacent segment disease – lumbar intraspinal synovial cysts in patients with osteoporosis: A case report

**DOI:** 10.1016/j.ijscr.2025.111690

**Published:** 2025-07-18

**Authors:** Lai-Zhou Ji, Ming-Zuo Guo, Si-Peng Li, Hao-Xuan Zhang

**Affiliations:** a Department of Orthopedic Surgery, The First Affiliated Hospital of Shandong First Medical University, NO16766 Jingshi Road, Jinan, Shandong Province 250014, China; b Department of Orthopedic Surgery, Shangdong Provincial Qianfoshan Hospital, Shandong University, NO16766 Jingshi Road, Jinan, Shandong Province 250014, China

**Keywords:** Adjacent segment disease, Case report, Lumbar intraspinal synovial cysts, Screw fracture, Waveflex semi-rigid rod

## Abstract

**Introduction:**

This paper presents a case of lumbar intraspinal synovial cysts (LISCs) associated with secondary osteoporosis, contributing to current understanding of LISC pathogenesis and offering a safe, effective surgical strategy tailored to complex clinical conditions.

**Case report:**

A 61-year-old woman with a history of rheumatoid arthritis and osteoporosis (lumbar spine T-score: −2.6) underwent posterior lumbar interbody fusion (PLIF) at the L4/5 segment for lumbar disc herniation and spondylolisthesis, as confirmed by MRI.

Ten months postoperatively, she developed a synovial cyst at the L3/4 segment, accompanied by a fracture of the L5 pedicle screw. Therefore, she underwent a revision surgery involving cyst excision, bone cement-reinforced screw fixation, and Waveflex semi-rigid rod implantation. The postoperative recovery was smooth, and no neurological abnormalities were found during the 9-month follow-up.

**Discussion:**

The surgical strategy effectively addressed three major challenges: excision of the cyst in the context of compromised spinal stability, revision fixation in osteoporotic bone using cement augmentation, and mitigation of adjacent segment degeneration (ASD) through motion-preserving Waveflex semi-rigid stabilization.

**Conclusion:**

The combination of cyst resection, Waveflex semi-rigid fixation, and cement-reinforced screw placement demonstrated favorable safety and efficacy in revision lumbar fusion for patients with secondary osteoporosis.

## Highlights

• Lumbar intraspinal synovial cysts are rare in osteoporotic patients.
• Diagnosis was confirmed by imaging and histopathology.
• The new internal fixation system reduced the loading of adjacent segments compared to conventional rigid titanium rods.
• Cement augmentation of the fractured pedicle screw increased pullout resistance.
• This hybrid approach combines cyst resection, Waveflex semi-rigid fixation, and cement-reinforced screw placement.

## 1 Introduction

Cysts located adjacent to the facet joints are referred to as juxta-facet cysts, encompassing both synovial and ganglion cysts. Synovial cysts represent a relatively uncommon form of cystic lesion within the spinal canal, originating from the synovial membrane of the facet joint [1]. While the optimal surgical approaches for LISCs remain debated, recent developments in dynamic stabilization systems, such as Waveflex rods (Medyssey), have demonstrated potential in relieving cyst-induced compression while preserving adjacent segment mobility. The present report introduces the novel application of Waveflex semi-rigid fixation combined with targeted bone grafting in a high-risk osteoporotic patient with post-fusion adjacent segment degeneration (ASD) and pedicle screw failure. This case report is reported in line with the SCARE criteria [2].

## 2 Case report

**Fig. 1:**
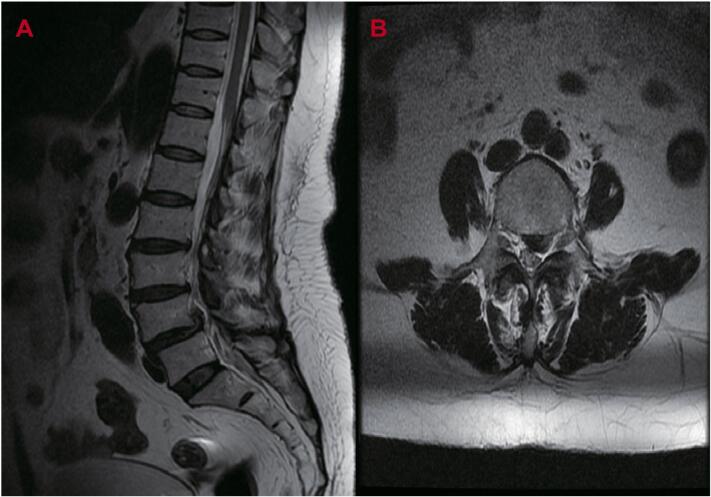
A-B. Preoperative Sagittal (A) and axial (B) MR images revealed intervertebral disc extrusion at the L4/5 segments towards the posterior and superior aspects, concurrent with spinal canal stenosis and left lateral recess stenosis.

A 61-year-old woman presented with lumbar and left leg pain persisting for 4 h prior to hospital admission, reporting a Visual Analog Scale (VAS) score of 8. Magnetic resonance imaging (MRI) scans (Fig. 1A/B) confirmed lumbar disc herniation and spondylolisthesis at the L4/5 level. The patient had a 20-year history of rheumatoid arthritis treated with Leflunomide and Prednisone, along with a 2-year history of osteoporosis. However, anti-osteoporotic medication had not been taken consistently. At admission, bone mineral density was 0.992 g/cm^2^, with a lumbar spine T-score of −2.6. The patient underwent posterior lumbar interbody fusion (PLIF) with pedicle screw fixation (6.0 mm × 45 mm) under general anesthesia, resulting in significant symptom relief and enabling a smooth discharge. Given the patient's age, the need for extensive decompression, and the likelihood of future revision surgery, rigid fixation was selected. Pedicle screws (6.0 mm × 45 mm) were used during the procedure, and postoperative pain was markedly reduced, with a VAS score of 1.

**Fig. 2:**
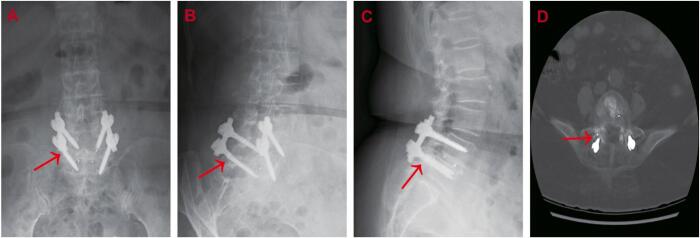
A-D. Postoperative anteroposterior (A), oblique (B) and lateral (C) radiographs and CT images (D) revealed the fracture of a screw (arrow).

Ten months after the initial fusion, the patient reported a sudden onset of lumbar pain radiating to both legs, with greater severity in the left leg and a VAS score of 9. Neurological assessments showed pain at 20° in the left leg and 30° in the right leg during the Lasegue test, along with positive Bragard signs bilaterally. Radiographs (Fig. 2A–C) and computed tomography (CT) images (Fig. 2D) identified a fracture of the right L5 pedicle screw.

**Fig. 3:**
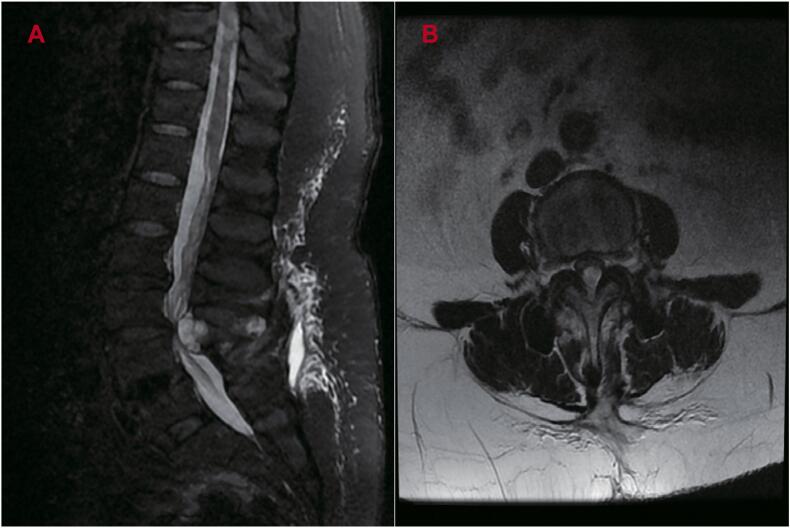
A-B. Sagittal (A) and axial (B) MR images showed lateral recess stenosis at the L3/4 segments secondary to synovial cysts.

MRI images (Fig. 3A/B) were obtained under a preliminary diagnosis of a space-occupying lesion within the spinal canal. Revision surgery was performed for decompression and Waveflex semi-rigid internal fixation across the L3 to L5 segments. The fractured pedicle screw on the right side of L5 was removed using a specialized extraction tool, and 1.5 mL of bone cement was injected into the original pedicle screw canal. Four pedicle screws (6.5 mm × 45 mm) were inserted through the right pedicles of L4 and L5 and the bilateral pedicles of L3. The pedicle screws at L4 and L5 were removed and replaced, while new screws were implanted at L3. Two Waveflex titanium rods (6.5 cm; Medyssey, Korea) were installed to replace the original rigid rods. To enhance fusion, the cortical bone of the bilateral transverse processes at L4/5 was decorticated using a bone drill, and autologous bone granules were grafted between the transverse processes, external to the titanium rods.

Intraoperative exploration identified a cystic lesion located on the dorsal side of the dural sac at the L3 segment, measuring approximately 3 cm × 1 cm × 1 cm. The lesion communicated with the facet joint and contained mucus, with a well-defined border, an intact capsule, and a firm texture. Adhesion to the dural sac was noted, along with compression of the dural sac and adjacent nerve roots.

**Fig. 4:**
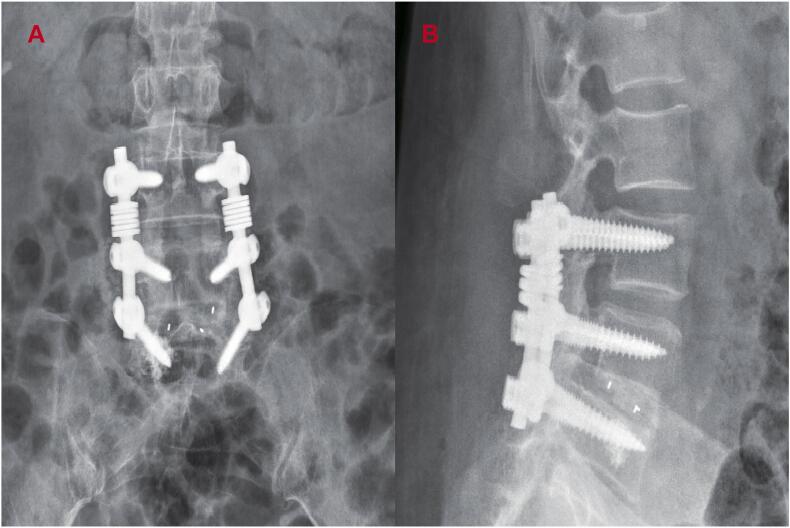
A-B. Anteroposterior (A) and lateral (B) radiographs after the spinal revision surgery showed correct positioning of the pedicle screws.

**Fig. 5:**
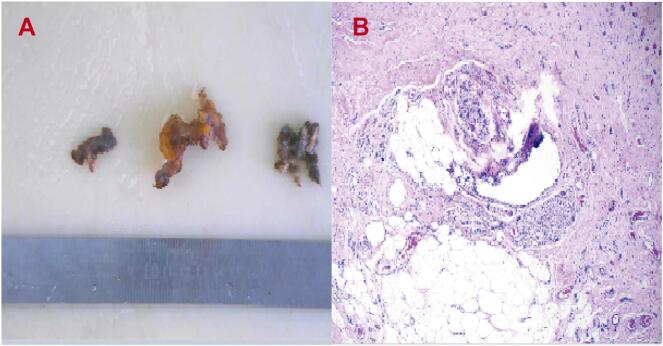
A-B. Pathological images (A) of the surgical specimen (B) revealed vascular fibrosis proliferation, partial necrotic cystic degeneration, and extensive infiltration of foamy cells.

Postoperative radiographs (Fig. 4A/B) confirmed accurate positioning of the pedicle screws. Tissue specimens were submitted to the Department of Pathology at Shandong Provincial Qianfoshan Hospital Institute for histopathological analysis (Fig. 5A/B). Following surgical intervention, the patient's symptoms promptly resolved, with a VAS score of 1. At the 9-month follow-up after revision surgery, no symptom recurrence or neurological deterioration was observed, and the Barthel index was recorded as 90.

## 3 Discussion

LISCs, which often present with neurological symptoms, were first described by Vosschulte and Borger in 1950 [3]. Although their exact etiology and pathogenesis remain under debate, many studies have linked their formation to factors such as degenerative disc disease, trauma, and rheumatoid arthritis. LISCs most commonly affect highly mobile spinal segments, particularly those in the lower lumbar region [4].

Increased segmental mobility frequently contributes to the development of facet joint osteoarthritis or degenerative lumbar spondylolisthesis (DLS), initiating a cycle of repetitive microtrauma and progressive degeneration. Over time, this cycle may lead to herniation of a weakened joint capsule, resulting in the formation of a synovial cyst. The occurrence of symptomatic LISCs following spinal fusion reflects a multifactorial process involving biomechanical stress, inflammatory activity, and metabolic imbalance. In the present case, the development of a synovial cyst at the L3/4 segment was likely attributed to increased motion at the adjacent segment following initial L4/5 fusion. Mechanical instability, in combination with chronic rheumatoid inflammation and glucocorticoid-induced osteoporosis, likely contributed to synovial membrane herniation.

Recent evidence indicates that patients with osteoporosis demonstrate a higher incidence of facet joint–derived cysts compared to the general population, likely due to accelerated capsular degeneration under repetitive shear stress [1,3]. Tobert et al. concluded that both intrinsic patient factors and biomechanical alterations following spinal fusion act synergistically to promote the development of ASD, including lumbar intraspinal synovial cysts [5,6].

The reported incidence of pedicle screw fracture ranges from 0.8 % to 24.6 %, with a significant increase observed in cases involving deformity corrections and multilevel fusion procedures [7]. Two primary factors contribute to screw failure following spinal fusion: insufficient integration of the bone graft with the vertebral body and a history of secondary osteoporosis combined with prolonged corticosteroid therapy.

A comprehensive assessment of the patient's clinical condition, surgical indications, degree of osteoporosis, and anticipated postoperative rehabilitation was conducted to systematically compare and analyze available surgical options, including simple decompression, rigid fixation extension, and alternative dynamic stabilization systems.

First, simple cystectomy was insufficient to address the underlying mechanical instability, particularly in the presence of a fractured L5 pedicle screw, which required revision of the failed internal fixation.

Second, rigid extension of fixation to the L3 vertebra would result in complete immobilization of the L3/4 segment, shifting mechanical stress to the L2/3 level and accelerating the progression of ASD. Additionally, patients with osteoporosis exhibit reduced tolerance to stress concentration generated by rigid fixation systems, resulting in a significantly increased risk of screw loosening during reoperation.

Third, the internal fixation system developed by Medyssey (South Korea) consists of three core components: titanium rods, pedicle screws, and screw plugs. The wave-shaped structure of the Waveflex titanium rods allows for controlled mobility at the L3/4 segment (10° flexion, 5° extension), thereby maintaining lumbar lordosis and preserving intervertebral disc height. Compared with elastic fixation systems such as Dynesys, the Waveflex system more closely replicates the physiological lordosis of the lumbar spine, significantly reducing load pressure on adjacent intervertebral discs, as demonstrated in biomechanical studies [[8], [9], [10], [11], [12], [13]]. Besides, the system's wave-shaped titanium rods demonstrated superior load distribution compared to traditional rigid constructs by absorbing axial forces that would otherwise concentrate at the L3/4 facet joints [8,14]. Disadvantages of semi-rigid fixation have been reported in the literature. Due to limited structural rigidity, it is not suitable for patients requiring spinal deformity correction, and the cost is higher than that of traditional rigid rods [15]. Table 1 summarizes the available literature about Waveflex Semi-Rigid System.

**Table 1:** Published studies on the efficacy of the Waveflex semi-rigid dynamic internal fixation system in the treatment of lumbar degenerative diseases.

Author (year)	Study type (patient)	Key findings
Kim DK. et al.(2016) [8]	Retrospective Cohort (*n* = 40)	Semirigid rods offer a similar or superior radiological and clinical efficacy compared to rigid rods during 24-month follow-up.
Gao W. et al. (2024) [9]	Retrospective Cohort (*n* = 50)	The Waveflex group demonstrated better outcomes in disc height index (DHI), intervertebral foramen height (IFH), and range of motion (ROM), along with superior overall spinal motor function parameters - including lumbar lordosis (LL), sacral slope (SS), and |pelvic incidence (PI)-LL| values - compared to the PLIF group at 1 year and 5 years postoperatively (*P* < 0.05).
Wu J. et al.(2014) [12]	Retrospective Cohort (*n* = 19)	Compared to the conventional fusion control group, the WavefleX dynamic stabilization system demonstrated significantly lower range of motion (ROM) at adjacent levels at both 6 months and 12 months postoperatively (P < 0.05).
Xue Y. et al. (2015) [13]	Retrospective Cohort (*n* = 26)	In all 26 patients undergoing Waveflex system fixation, adjacent segment disc space height (DSH) significantly increased (P < 0.05) and range of motion (ROM) significantly decreased (P < 0.05) compared to preoperative values at 7 days, 6 months, and the final follow-up, with no implant failures observed.
Zhang Z. et al.(2025) [14]	Retrospective Cohort (*n* = 31)	Visual Analogue Scale (VAS) and Oswestry Disability Index (ODI) scores demonstrated significant improvement (P < 0.05) at 3 months, 6 months, and final follow-up compared with the preoperative period.

Fourth, cement augmentation of the fractured L5 pedicle screw enhanced pullout resistance and effectively reduced the risk of fixation failure in osteoporotic bone. Previous studies have demonstrated a positive correlation between the fixation strength of cement-augmented pedicle screws and the severity of osteoporosis [16].

## 4 Conclusion

The hybrid approach, which integrates dynamic stabilization with selective rigid fixation, has marked a significant shift in the management of post-fusion complications, particularly in situations where conventional revision techniques have been shown to accelerate adjacent segment degeneration.

## Author contribution

LZJ participated in the drafting and writing of the manuscript; LZJ, MZG and SPL participated in the design of the study and performed the operation; HXZ conceived the study and helped to draft the manuscript.

## Informed consent

Written informed consent was obtained from the patient for publication and any accompanying images. A copy of the written consent is available for review by the Editor-in-Chief of this journal on request.

## Guarantor

Hao-Xuan Zhang.

## Declaration of Generative AI and AI-assisted technologies in the writing process

In the preparation of this manuscript, a generative AI tool “DeepSeek” was employed exclusively to assist with grammar and syntax correction. The application of this tool did not influence the research design, data analysis, or interpretation of results. All scientific content, insights, and conclusions presented herein are solely the responsibility of the authors. Every AI-generated correction was carefully reviewed and validated by the authors to ensure that the integrity and accuracy of the content were maintained.

## Funding declaration

This study was supported by grants from the Taishan Scholar Program of Shandong Province (No.tsqn202312357) and National Science Foundation of China (No.81902188).

## Ethical approval of studies

No ethics committee approval is required at our institution for a case report involving a single patient.

## Declaration of competing interest

The authors declare that they have NO affiliations with or involvement in any organization or entity with any financial interest in the subject matter or materials discussed in this manuscript.

## References

[1] Kim H.S., Kim S.W. (2017). Hemorrhagic lumbar synovial cyst after microscopic discectomy. Korean J Spine..

[2] Revised Surgical CAse REport (SCARE) Guideline: An Update for the age of Artificial Intelligence - Premier Science. https://premierscience.com/pjs-25-932/.

[3] Vosschulte K., Borger G. (1950). The inflammatory processes in sciatica due to intervertebral disk hernia. Med Monatsschr..

[4] Khan A.M., Girardi F. (2006). Spinal lumbar synovial cysts. Diagnosis and management challenge. Eur. Spine J..

[5] Tobert D.G., Antoci V., Patel S.P., Saadat E., Bono C.M. (2017). Adjacent segment disease in the cervical and lumbar spine. Clinical Spine Surgery..

[6] McDonald C.L., Alsoof D., Glueck J. (2023). Adjacent segment disease after spinal fusion. JBJS Rev..

[7] Secer M., Polat O., Cinar K., Ulutas M. (2018). A simple technique for removing broken pedicle screws. Turkish Neurosurg..

[8] Kim D.K., Lim H., Rim D.C., Oh C.H. (2016). Clinical and Radiological Comparison of Semirigid (WavefleX) and Rigid System for the Lumbar Spine. Korean J Spine..

[9] Gao W., Wang X., Chen Y. (2024). Long-term efficacy of Waveflex semi-rigid-dynamic-internal-fixation system in delaying intervertebral disc degeneration at adjacent segments and improving spinal sagittal imbalance. Sci. Rep..

[10] Bernhardt M., Bridwell K.H. (1989). Segmental analysis of the sagittal plane alignment of the normal thoracic and lumbar spines and thoracolumbar junction. Spine (Phila Pa 1976).

[11] Kim W.J., Ma C.H., Kim S.H. (2019). Prevention of adjacent segmental disease after fusion in degenerative spinal disorder: correlation between segmental lumbar lordosis ratio and pelvic incidence-lumbar lordosis mismatch for a minimum 5-year follow-up. Asian Spine J..

[12] Wu J., Du J., Jiang X. (2014). Analyses of segment motor function in patients with degenerative lumbar disease on the treatment of WavefleX dynamic stabilization system. Zhonghua Yi Xue Za Zhi.

[13] Xue Y., Wang D., Dai W., Ma C., Xia J. (2015). Medium-term effectiveness of waveflex system in treatment of multiple lumbar degenerative diseases. Zhongguo Xiu Fu Chong Jian Wai Ke Za Zhi..

[14] Zhang Z., Shao J., Su N. (2025). The clinical efficacy of hybrid surgery based on the Waveflex semi-rigid dynamic internal fixation system for the treatment of lumbar degenerative diseases: over three-year follow-up study. BMC Musculoskelet. Disord..

[15] Qi L., Li M., Zhang S. (2013). Comparative effectiveness of PEEK rods versus titanium alloy rods in lumbar fusion: a preliminary report. Acta Neurochir (Wien).

[16] Saadeh Y.S., Swong K.N., Yee T.J. (2020). Effect of fenestrated pedicle screws with cement augmentation in osteoporotic patients undergoing spinal fusion. World Neurosurgery.

